# Clinical and cost-effectiveness of oral sodium bicarbonate therapy for older patients with chronic kidney disease and low-grade acidosis (BiCARB): a pragmatic randomised, double-blind, placebo-controlled trial

**DOI:** 10.1186/s12916-020-01542-9

**Published:** 2020-04-09

**Authors:** The BiCARB study group, Miles D. Witham, Margaret Band, Aimun Ahmed, Michael K. Almond, Gowrie Balasubramaniam, Kolitha Basnayake, Deepak Bhatnagar, Anthony Chan, Huey Yi Chong, Peter T. Donnan, Neill Duncan, Geeta Hampson, May Khei Hu, Philip A. Kalra, Gwen Kennedy, Adam Kirk, Edmund J. Lamb, Stewart Lambie, Roberta Littleford, Paul McNamee, Biswa Mishra, Sandip Mitra, Johann Nicholas, Deirdre Plews, Petra Rauchhaus, Roy L. Soiza, Paul E. Stevens, Deepa Sumukadas, Wai Tse, Graham Warwick, Martin Wilkie, Georgia Winnett, Alison Avenell

**Affiliations:** AGE Research Group, NIHR Newcastle Biomedical Research Centre, Biomedical Research Building, Campus for Ageing and Vitality, Newcastle, NE4 5PL UK

**Keywords:** Sodium bicarbonate, Renal insufficiency, chronic, Acidosis, Randomised controlled trial

## Abstract

**Background:**

Chronic kidney disease with metabolic acidosis is common in older people, but the effectiveness of oral sodium bicarbonate therapy in this group is unclear. We tested whether oral sodium bicarbonate provides net health benefit for older people with advanced chronic kidney disease and serum bicarbonate concentrations < 22 mmol/L.

**Methods:**

Pragmatic multicentre, parallel group, double-blind, placebo-controlled randomised trial. We recruited adults aged ≥ 60 years with estimated glomerular filtration rate of < 30 mL/min/1.73 m^2^, not receiving dialysis, with serum bicarbonate concentration < 22 mmol/L, from 27 nephrology and geriatric medicine departments in the UK. Participants received oral sodium bicarbonate (up to 3 g/day) or matching placebo given for up to 2 years, randomised in a 1:1 ratio. The primary outcome was between-group difference in the Short Physical Performance Battery (SPPB) at 12 months, adjusted for baseline values, analysed by intention to treat. Secondary outcomes included generic and disease-specific quality of life (EQ-5D and KDQoL tools), anthropometry, renal function, walk distance, blood pressure, bone and vascular health markers, and incremental cost per quality-adjusted life year gained.

**Results:**

We randomised 300 participants between May 2013 and February 2017, mean age 74 years, 86 (29%) female. At 12 months, 116/152 (76%) participants allocated to bicarbonate and 104/148 (70%) allocated to placebo were assessed; primary outcome data were available for 187 participants. We found no significant treatment effect for the SPPB: bicarbonate arm 8.3 (SD 2.5) points, placebo arm 8.8 (SD 2.2) and adjusted treatment effect − 0.4 (95% CI − 0.9 to 0.1, *p* = 0.15). We found no significant treatment effect for glomerular filtration rate (0.6 mL/min/1.73 m^2^, 95% CI − 0.8 to 2.0, *p* = 0.39). The bicarbonate arm showed higher costs and lower quality of life as measured by the EQ-5D-3L tool over 1 year (£564 [95% CI £88 to £1154]); placebo dominated bicarbonate under all sensitivity analyses. Adverse events were more frequent in those randomised to bicarbonate (457 versus 400).

**Conclusions:**

Oral sodium bicarbonate did not improve physical function or renal function, increased adverse events and is unlikely to be cost-effective for use by the UK NHS for this patient group.

**Trial registration:**

European Clinical Trials Database (2011-005271-16) and ISRCTN09486651; registered 17 February 2012.

## Background

Chronic kidney disease (CKD) becomes increasingly common with older age, with approximately 2% of the population aged 70 years and over suffering from advanced (estimated glomerular filtration rate [eGFR] < 30 mL/min/1.73 m^2^) chronic kidney disease [1]. Impaired ability to excrete hydrogen ions means that advanced CKD is accompanied by metabolic acidosis in approximately 20% of cases, with rates higher at lower levels of renal function [2]. Metabolic acidosis has been associated in observational studies with a range of adverse health outcomes in patients with CKD, including worse cardiovascular health, lower bone mineral density and increased fracture risk, impaired muscle function and more rapid progression of kidney disease [3–8]. The extent to which acidosis causes these phenomena remains unclear.

Oral sodium bicarbonate has been used for decades to counteract metabolic acidosis. Few trials have tested whether sodium bicarbonate is effective at preventing adverse outcomes from advanced CKD, and is safe in the context of increased sodium load, as opposed to merely increasing the concentration of circulating bicarbonate. Two recent systematic reviews and meta-analyses of small trials of moderate quality suggested a modest beneficial effect on estimated GFR and serum bicarbonate, an uncertain effect on progression to end-stage kidney disease and contrasting effects on blood pressure (no effect in one review, and a small increase in the risk of hypertension in the other), and found no data on the effect of bicarbonate on physical function or quality of life [9, 10]. The mean age of participants in included trials was young, ranging from 41 to 65 years. Sodium bicarbonate carries risks of gastrointestinal side effects, the large tablets and large number of tablets required are awkward for patients to take long term, and there are concerns that the sodium content might increase blood pressure or circulatory overload. These issues are of particular relevance for older people, who make up the majority of people in the UK with advanced kidney disease and are more likely to suffer side effects due to coexisting multimorbidity and polypharmacy. Current guidelines for the management of chronic kidney disease recommend using oral sodium bicarbonate to treat metabolic acidosis but acknowledge the dearth of evidence in this area [11, 12].

A focus on single disease outcomes may not always be appropriate for older people with multimorbidity; improvements in a single organ domain may be counterbalanced by harms in other systems. Older people consistently rank physical function and quality of life as the most important outcomes [13] and measurement of these outcomes allows the impact of an intervention to be integrated across multiple organ systems. The primary objective of the BiCARB trial was to determine whether oral bicarbonate therapy improves physical function compared to placebo in older people with CKD and mild acidosis. The secondary objectives were to assess the effect of bicarbonate supplementation on health-related quality of life, biochemical markers of CKD, bone and vascular health, adverse events and healthcare costs.

## Methods

### Study design

We conducted a parallel group, double-blind, placebo-controlled randomised trial. The trial was designed in response to a commissioning brief issued by the National Institute for Health Research (NIHR) Health Technology Assessment board. We recruited participants from nephrology and geriatric medicine outpatients at 27 UK hospitals. Ethical approval was granted by the East of Scotland NHS Research Ethics Committee (approval 12/ES/0023); the trial was also approved by the UK Medicines and Healthcare Regulatory Authority (EudraCT number 2011-005271-16; Clinical Trial Authorisation number 41692/0001/001-0001). The trial was co-sponsored by the University of Dundee and NHS Tayside (Tayside Academic Health Sciences Collaboration). The trial was registered at www.isrctn.com (ISRCTN09486651). The protocol has been published [14].

### Participants

Participants were eligible for inclusion if they were ≥ 60 years with advanced CKD (estimated GFR < 30 mL/min/1.73 m^2^ by Modification of Diet in Renal Disease study four variable (MDRD4) equation [15]), not receiving dialysis, with serum bicarbonate concentrations < 22 mmol/L. No lower limit on bicarbonate concentration was stipulated. The MDRD4 equation was used as this was the equation in common use in the UK at the time that the trial was designed. We excluded participants if they were currently taking bicarbonate (such participants could however enter a 3-month washout period, after which they became eligible), had a diagnosis of renal tubular acidosis, were taking a bisphosphonate, were terminally ill, could not give written informed consent, had uncontrolled hypertension (blood pressure > 150/90 despite four or more antihypertensive agents) or decompensated chronic heart failure, were participating in another clinical trial, or were allergic to sodium bicarbonate tablets or lactose (excipient in the tablets). All participants gave written informed consent.

We relaxed the exclusion criteria for the trial part-way through recruitment, both to improve slow recruitment rates, in part influenced by the lack of equipoise amongst clinicians and to better reflect current UK clinical practice. We reduced the lower age limit from 65 to 60; we included patients taking calcium acetate or sevelamer; we included patients with blood pressure controlled on home readings even if office blood pressure was high, and we allowed those currently taking bicarbonate to undergo a 3-month washout period with assessment of eligibility at the end of the washout period.

### Randomisation and masking

We randomised participants using an interactive web-based randomisation, drug assignment and inventory management system (TRuST) run by the Health Informatics Centre, University of Dundee. The system was run independently from the research team to preserve allocation concealment. We performed randomisation in a 1:1 ratio, stratified by site, and employed a minimisation algorithm to balance male vs female sex, CKD eGFR category 4 vs category 5, and age < 75 years versus ≥ 75 years. We allocated study medication bottles to participants (one bottle per month) with either 500 mg sodium bicarbonate tablets or matching placebo tablets; we allocated bottles using identification numbers generated by the TRuST randomisation system, and bottles carried no external indication to identify to which trial arm participants were allocated. Participants, researchers and clinical teams and researchers including the statistician remained masked to treatment allocation until after completion of the main trial analysis.

### Trial intervention and comparator

Active and placebo tablets were manufactured and bottled by Legosan AB (Kumla, Sweden). For the first 3 months of participation, we asked participants to take one tablet three times daily (i.e. 500 mg of sodium bicarbonate or placebo three times a day). We measured serum bicarbonate concentration at the 3-month visit to guide dose titration. If bicarbonate was < 22 mmol/L, we asked participants to increase their study medication to two tablets three times a day (i.e. 1 g of sodium bicarbonate or placebo three times a day). If bicarbonate was ≥ 22 mmol/L, we asked participants to continue taking one tablet three times a day for the remainder of the trial. Although higher doses of bicarbonate have been used in some previous trials [9], we selected this dose to reflect current UK clinical practice in the treatment of acidosis; we judged that higher doses would be poorly tolerated by older people on multiple medications. No trial-specific dietary advice beyond usual care was given, and no specific recommendations on management of low serum bicarbonate concentrations were given to study physicians.

### Outcomes

We measured outcomes at baseline and 3, 6, 12 and 24 months. The primary outcome was the between-group difference in the Short Physical Performance Battery (SPPB) at 12 months, adjusted for baseline values. The SPPB is a 12-point measure of lower limb strength and balance, with higher values denoting better function, that predicts future disability, need for care and death [16–18]. The minimum clinically important difference has been derived for the measure [16].

Secondary outcome measures were generic (EuroQoL EQ-5D-3L) [19] and disease-specific (Kidney Disease Quality of Life) [20] health-related quality of life, anthropometry (weight, mid-arm muscle circumference, triceps skinfold thickness, mid-thigh circumference), physical performance (6-min walk speed, grip strength) and renal function (serum creatinine, cystatin C, and urinary albumin/creatinine ratio); markers of bone turnover and mineral metabolism (serum calcium, phosphate, bone-specific alkaline phosphatase, tartrate-resistant acid phosphatase 5b, 25-hydroxyvitamin D and 1,25 dihydroxyvitamin D); vascular health (blood pressure, B-type natriuretic peptide and serum cholesterol); and relevant markers, including haemoglobin, thyroid-stimulating hormone and albumin. Originally planned substudies examining arterial stiffness and bone mineral density were discontinued due to very low recruitment rates. We recorded all adverse events, including commencement of renal replacement therapy (defined as the first episode of haemodialysis, peritoneal dialysis or renal transplantation). We recorded falls prospectively using participant-completed fall diaries, and we measured adherence by counting pills taken divided by the number of pills expected to be taken. For the health economic analysis, we collected information on health and social care use using participant-completed questionnaires at follow-up visits, and we combined these data with health-related quality of life measures (EQ-5D-3L and ICEpop CAPability measure for Older people [ICECAP-O]) and a measure of global life satisfaction to derive the incremental cost per quality-adjusted life year (QALY) gained.

### Statistical analysis

We based the original sample size calculation on the ability to detect a 1-point difference in the primary outcome (SPPB), a difference proposed as the minimum clinically important difference (MCID) by previous investigators [16]. Previous work with older people has shown a standard deviation of 2.6 for the SPPB [21]. To detect a 1-point difference between groups at 12 months with 90% power given this standard deviation would require 143 participants per group given a two-sided alpha of 0.05. To ensure that the trial had sufficient power for the key secondary outcome of health-related quality of life, we also estimated the sample size required to detect the MCID for the EQ-5D-3L measure. For the EQ-5D-3L, we assumed a MCID of 0.074 based on previous data [22]. To detect this change at a two-sided alpha of 0.05 and power of 90% power, assuming a SD of change of 0.2 as found in our previous studies [23, 24] would require 154 participants per group. Assuming a 10% loss to follow-up every 6 months (based on previous medication trials in frail older people) [24], we estimated that we would require 380 patients (190 per group) to detect the MCID with 90% power for the primary outcome and the EQ-5D-3L at 12 months.

We used a prespecified statistical analysis plan. All analyses were by intention to treat using SAS v9.4 (SAS Institute Inc., Cary, NC, USA). We analysed the primary outcome (between-group difference in the SPPB at 12 months) using linear mixed models, adjusted for baseline measurements, minimisation variables (age, sex and CKD category) and a random effect variable for recruitment site. We analysed secondary outcomes using repeated measures mixed models, including all participants and including data from all available timepoints. Models were adjusted for baseline values and the minimisation variables. We conducted time-to-event analyses (time to death, time to commencing renal replacement therapy) using Cox proportional hazards models adjusted for minimisation variables. All participants were included in these analyses, with participants censored at the point of dropout or truncation of follow-up for those not reaching the analysis endpoint before 24 months. For all analyses, we took a two-sided *p* value of < 0.05 as significant with no adjustment for multiple testing. For the health economic analysis, we undertook a cost-utility analysis which involved estimation of the incremental costs and incremental effects (QALYs) from a health and social care perspective. We used UK sources for unit cost estimates for items of resource use [25, 26] but did not include costs of medications other than bicarbonate. We derived estimates using generalised linear regression modelling, with adjustment for skewed data and for baseline differences in cost, EQ-5D-3L and minimisation variables. We used non-parametric bootstrap methods to calculate confidence intervals around cost and QALY differences. We generated cost-effectiveness acceptability curves to show the probability that bicarbonate therapy was cost-effective for different values of willingness to pay per additional QALY. We conducted a series of sensitivity analyses, using different measures of quality of life to derive QALYs, testing a range of different assumptions on costs and using multiple imputation to account for missing data. Sensitivity analyses included analyses that included renal replacement therapy costs assumed to be incurred after dropout from the trial for those who started renal replacement therapy but then discontinued trial participation.

An independent Data Monitoring Committee (DMC) met every 6 months to review unmasked reports. The DMC examined trial safety, data quality and study conduct and provided advice to the Sponsor, Trial Steering Committee (TSC) and funder on the appropriateness of continuing the trial. No interim efficacy analyses were planned or conducted. The independent TSC recommended closure of recruitment to the trial after 300 participants had been randomised, based on a review of declining recruitment rates and informed by revised sample size calculations that showed that a sample size of 300 had 87% power to detect a 1-point difference in the primary outcome of the short physical performance battery. The independent TSC also recommended that follow-up be truncated after the final 12-month (primary outcome) visit for the last participant to facilitate earlier dissemination of results. The decision to close the trial to further recruitment was made by the trial funder.

## Results

**Fig. 1:**
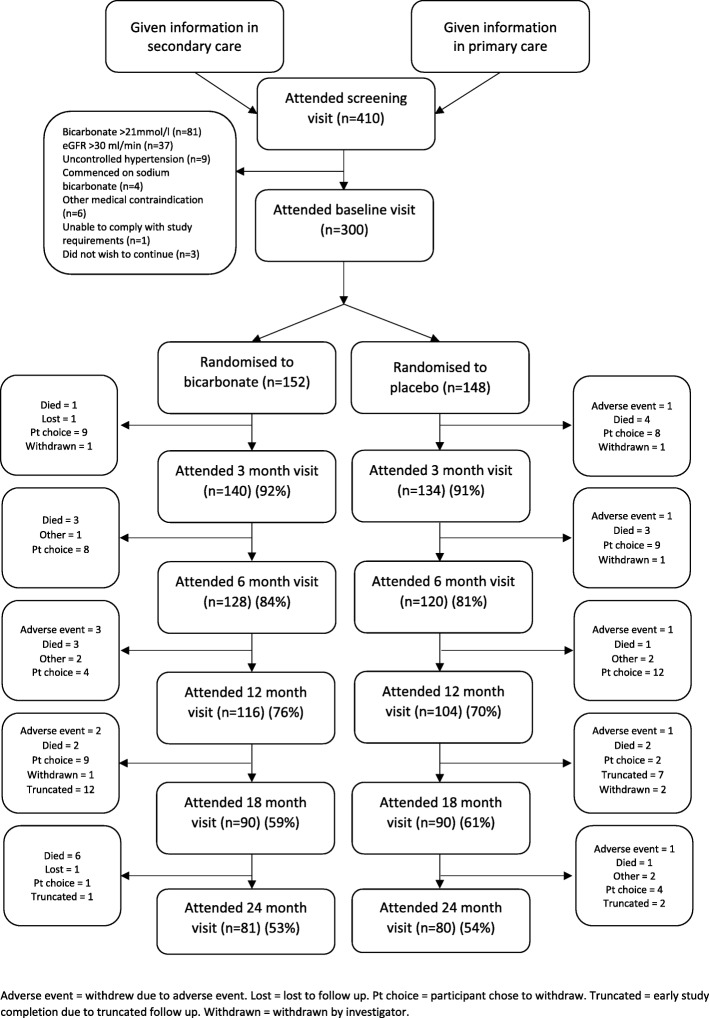
CONSORT diagram for participant flow through the trial

We randomised 300 participants into the trial between 1 May 2013 and 28 February 2017. Figure 1 depicts the flow of participants through the trial; the mean length of follow-up per participant was 16.4 months (16.6 (SD 8.9) months in the bicarbonate arm (median 24, IQR 12 to 24 months) and 16.3 (SD 9.4) months in the placebo arm (median 24, IQR 6 to 24 months)). Data for the primary outcome analysis were available for 274 participants at baseline (140 in the bicarbonate arm and 134 in the placebo arm) and for 187 participants at 12 months (97 in the bicarbonate arm and 90 in the placebo arm). One hundred sixty-one participants completed the full 24-month follow-up period. Loss to follow-up was due to a combination of deaths and illness; most participants who commenced dialysis chose to withdraw from the trial due to the added burden of dialytic treatment, although this was not mandated by the protocol. Twenty-two participants had their follow-up truncated due to termination of the trial.

**Fig. 2:**
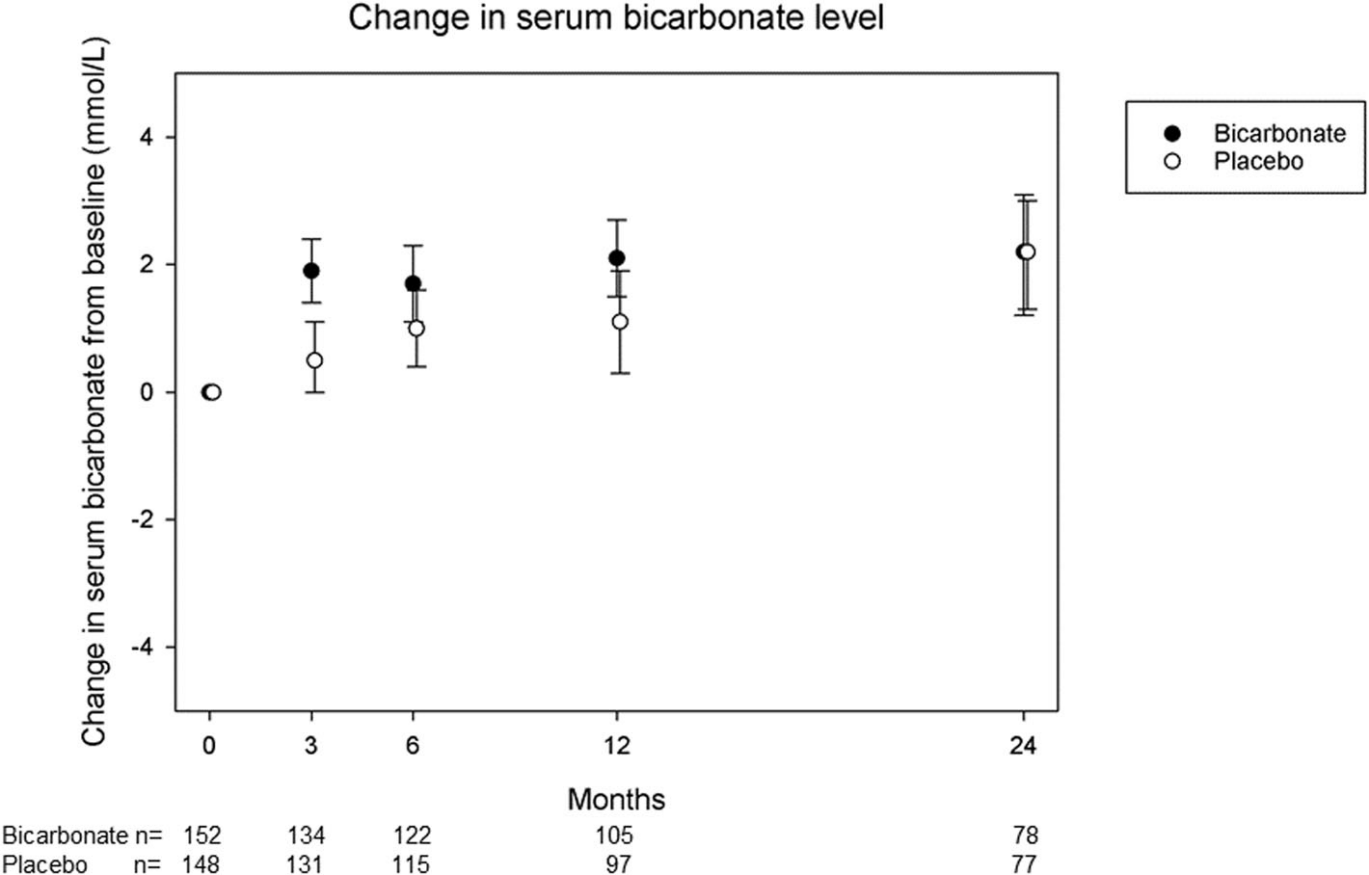
Serum bicarbonate concentrations. Values are mean and 95% CI

Baseline details for randomised participants in both arms of the trial are given in Table 1. Only four participants underwent the 3-month washout option prior to randomisation. Adherence by tablet count was moderate: 73% (95% CI 67 to 78) in the bicarbonate arm and 73% (95% CI 67 to 80) in the placebo arm. Forty-six of 152 participants in the bicarbonate arm had a serum bicarbonate concentration of < 22 mmol/L and were uptitrated at the 3-month visit, compared to 83/148 in the placebo arm. A modest but significant increase in serum bicarbonate concentration of 1.7 mmol/L (95% CI 1.0 to 2.4, *p* < 0.001) was seen in the intervention arm compared to placebo at 3 months; this difference attenuated with time and was no longer significant by 24 months, as shown in Fig. 2. During the course of the trial, 18 participants in the placebo group stopped taking study medication on the advice of the clinical team in order to start taking prescribed sodium bicarbonate. These individuals were retained in follow-up and analysed as per their initial randomised allocation.

**Table 1:** Baseline characteristics of randomised participants (*n* = 300)

	Sodium bicarbonate (*n* = 152)	Placebo (*n* = 148)
Mean age (years) (SD)	73.9 (7.6)	74.0 (6.6)
Age 60–64 (%)	13 (8.6)	13 (8.8)
Age 65–69 (%)	44 (28.9)	22 (14.9)
Age 70–74 (%)	23 (15.1)	44 (29.7)
Age 75–79 (%)	30 (19.7)	37 (25.0)
Age 80–84 (%)	32 (21.1)	24 (16.2)
Age 85 and over (%)	10 (6.6)	8 (5.4)
Female sex (%)	42 (27.6)	44 (29.7)
Ethnicity		
White (%)	144 (94.7)	143 (96.6)
East Asian (%)	0 (0)	1 (0.7)
Black (%)	1 (0.7)	0 (0)
South Asian (%)	4 (2.6)	2 (1.4)
Hispanic (%)	1 (0.7)	0 (0)
Other (%)	2 (1.3)	2 (1.4)
Cause of renal dysfunction*		
Hypertension (%)	37 (24.3)	40 (27.0)
Diabetes mellitus (%)	23 (15.1)	23 (15.5)
Glomerulonephritis (%)	9 (5.9)	11 (7.4)
Polycystic kidney disease (%)	11 (7.2)	9 (6.1)
Vascular disease (%)	19 (12.5)	21 (14.2)
Other (%)	52 (34.2)	63 (42.6)
Not known (%)	31 (20.4)	22 (14.9)
Cardiovascular comorbidity		
Hypertension (%)	135 (88.8)	129 (87.2)
Diabetes mellitus (%)	54 (35.5)	47 (31.8)
Ischaemic heart disease (%)	26 (17.1)	31 (20.9)
Stroke (%)	16 (10.5)	12 (8.1)
Heart failure (%)	19 (12.5)	5 (3.4)
Peripheral vascular disease (%)	14 (9.2)	10 (6.8)
Previous fragility fracture (%)	2 (1.3)	5 (3.4)
Mean number of medications (SD)	8.2 (3.7)	7.9 (3.3)
Medication use:		
ACEi/ARB (%)	105 (69.1)	91 (61.5)
Phosphate binder (%)	32 (21.1)	28 (18.9)
Activated vitamin D (%)	77 (50.7)	73 (49.3)
Erythropoietin (%)	89 (58.6)	106 (71.6)
Iron (%)	60 (39.5)	51 (34.5)
Mean eGFR (mL/min/1.73 m^2^) (SD)	19.7 (6.5)	18.2 (6.4)
CKD category 5 (%)	34 (22.4)	48 (32.4)
Mean serum bicarbonate (mmol/L) (SD)	20.6 (2.6)	20.1 (2.5)
Mean haemoglobin (g/L) (SD)	115 (14)	117 (17)
Mean serum potassium (mmol/L) (SD)	4.9 (0.5)	4.9 (0.5)
Mean Short physical performance battery (SD)	8.0 (2.4)	8.1 (2.2)
Mean 6-min walk distance (m) (SD)	304 (134)	317 (133)
Mean handgrip strength (kg) (SD)		
Males	26.6 (8.8)	28.0 (7.6)
Females	15.4 (4.8)	15.8 (4.4)
Mean body mass index (kg/m^2^) (SD)	28.9 (4.5)	28.3 (4.6)
Mean mid-arm muscle circumference (cm) (SD)	24.9 (3.6)	24.8 (4.0)
Mean triceps skinfold thickness (mm) (SD)	16 (8)	17 (9)
Mean mid-thigh circumference (cm) (SD)	47.4 (7.0)	46.8 (7.0)
Mean EQ-5D-3L (SD)	0.73 (0.22)	0.74 (0.24)
Mean EQ-5D thermometer (SD)	69 (19)	71 (19)
Mean KDQoL scores		
SF36 PCS (SD)	36 (11)	36 (11)
SF36 MCS (SD)	53 (11)	54 (9)
Burden (SD)	75 (26)	75 (25)
Symptoms (SD)	79 (14)	81 (12)
Effects (SD)	86 (14)	87 (15)
Mean office systolic blood pressure (mmHg) (SD)	143 (18)	143 (18)
Mean office diastolic blood pressure (mmHg) (SD)	75 (11)	73 (10)

*ACEi* angiotensin-converting enzyme inhibitor, *ARB* angiotensin receptor blocker, *CKD* chronic kidney disease, *KDQOL* Kidney Disease Quality of Life questionnaire, *SF-36* Short-Form 36-Item questionnaire, *PCS* physical component summary, *MCS* mental component summary, *EQ-5D* EuroQoL 5 dimension quality of life questionnaire

*More than one aetiology possible; thus, values sum to > 100%

There was no significant between-group difference in the primary outcome (SPPB at 12 months, adjusted for baseline values, age, sex and CKD stage). The adjusted treatment effect was − 0.4 points (95% CI − 0.9 to 0.1, *p* = 0.15) (negative values indicate worse physical performance in the intervention arm); analysis adjusted only for baseline SPPB gave the same result. Multiple imputation to account for missing data gave similar results (adjusted treatment effect − 0.3 points, 95% CI − 1.0 to 0.3, *p* = 0.29), and repeated measures analysis across the whole of trial follow-up gave a treatment effect of − 0.6 points (95% CI − 1.0 to − 0.1, *p* = 0.02). The participants who were unable to perform the SPPB at 12 months had borderline lower baseline SPPB scores, lower baseline grip strength and lower 6-min walk distance in both the bicarbonate and placebo arms but had similar baseline serum bicarbonate concentrations. Full details are given in Additional File 1.

**Fig. 3:**
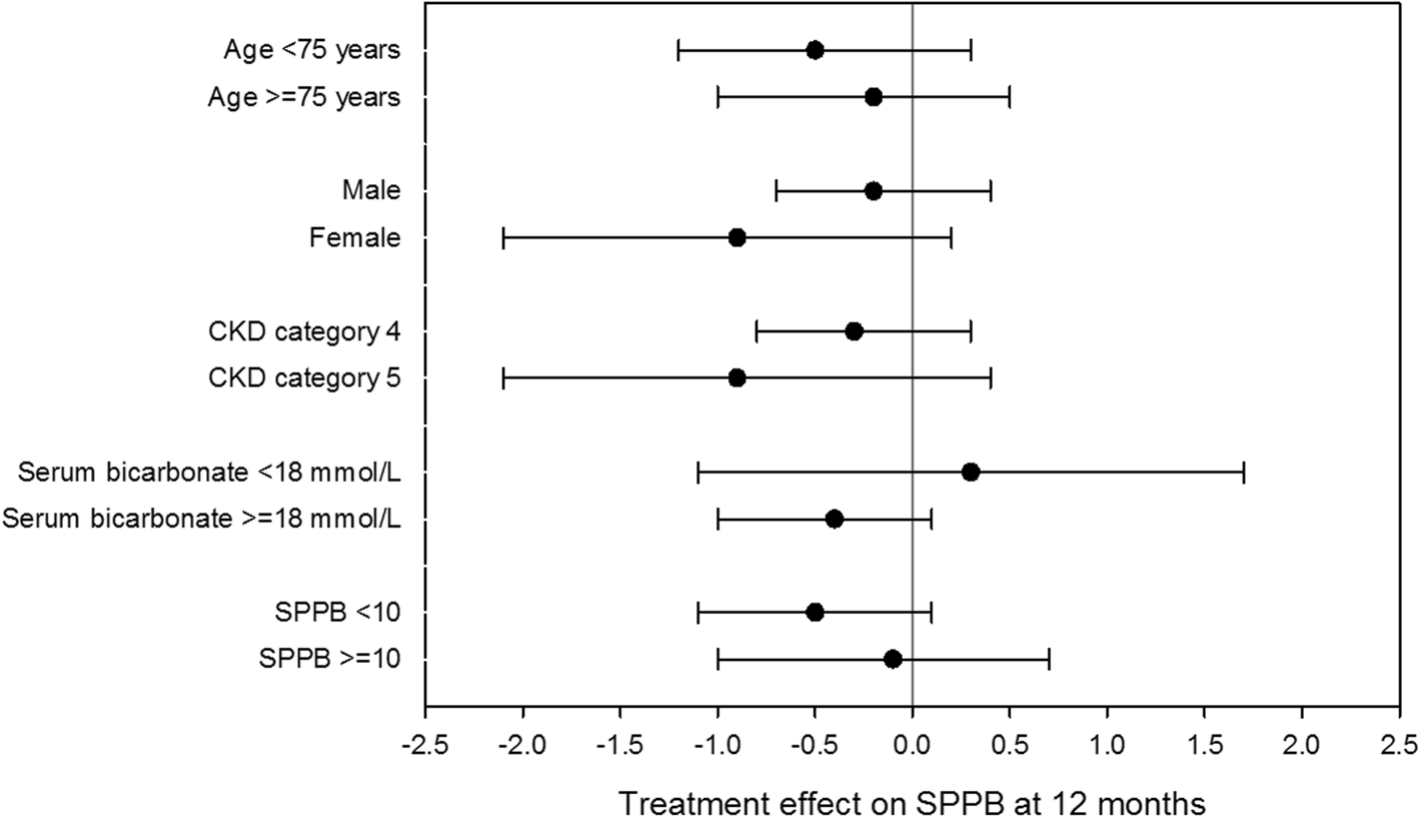
Subgroup analyses for the primary outcome (Short Physical Performance Battery). Values are mean and 95% CI

We conducted prespecified subgroup analyses for the primary outcome by age, sex, baseline eGFR category, baseline serum bicarbonate concentration and baseline SPPB score; results are given in Fig. 3. No significant treatment by subgroup interactions were found. We conducted a further pre-planned subgroup analysis, comparing the primary outcome treatment effect for those with good adherence to study medication (defined a priori as > 80%) versus poorer adherence to study medication (defined a priori as ≤ 80%). Those with good adherence showed an adjusted treatment effect at 12 months of − 0.6 (95% CI − 1.4 to 0.1; *p* = 0.07), compared to an adjusted treatment effect of 0.0 (95% CI − 0.7 to 0.7; *p* = 0.97) for those with poorer adherence. The difference in treatment effects was not significant (*p* for interaction = 0.27).

Adjusted treatment effects for secondary outcomes are given in Table 2. A total of 66/300 (22%) of participants commenced dialysis or underwent renal transplantation during the trial, with no difference between the bicarbonate and placebo arms (33 vs 33; *p* = 1.0). Time to event analysis showed no significant difference in the risk of commencing renal replacement therapy (HR 1.22, 95% CI 0.74 to 2.02; *p* = 0.43) (values greater than 1 indicate higher risk in the intervention arm), or for reaching a composite outcome of time to either doubling of serum creatinine, a 40% reduction in eGFR, or commencing renal replacement therapy (HR 1.16, 95% CI 0.73 to 1.84; *p* = 0.53). More participants in the bicarbonate arm reported at least one fall than in the placebo arm but this did not reach significance (49 vs 39; *p* = 0.26); the incident rate in each arm was not significantly different (bicarbonate arm, 0.99 per year [95% CI 0.61 to 1.38]; placebo arm, 0.72 per year [95% CI 0.25 to 1.19]; *p* = 0.38). Median time to first fall amongst those who fell was shorter in the bicarbonate arm (130 days vs 194 days). Cox proportional hazards modelling of time to first fall, adjusted for age, sex and CKD category, showed a HR of 1.43, 95% CI 0.94 to 2.20, *p* = 0.09. Fragility fracture numbers were low: 5/152 in the bicarbonate arm and 2/148 in the placebo arm.

**Table 2:** Secondary outcomes—adjusted treatment effects (repeated measures analyses using data from all available timepoints, adjusted for age, sex and CKD category)

	Treatment effect (bicarbonate–placebo) (95% CI)	*p*
Physical function and anthropometry		
Six-min walk distance (m)	− 33 (− 62 to − 4)	0.02
Grip strength (kg)	− 1.5 (− 2.8 to − 0.2)	0.03
Weight (kg)	0.2 (− 2.9 to 3.4)	0.89
Mid-arm muscle circumference (cm)	0.0 (− 0.6 to 0.6)	0.99
Triceps skinfold thickness (mm)	− 1 (− 2 to 1)	0.34
Mid-thigh circumference (cm)	0.1 (− 0.8 to 1.1)	0.80
Quality of life		
EuroQoL EQ-5D-3L	− 0.04 (− 0.08 to 0.00)	0.06
EuroQoL EQ-5D visual analogue scale	− 3 (− 7 to 1)	0.09
KDQOL symptoms	− 1 (− 3 to 2)	0.67
KDQOL burden of disease	− 3 (− 8 to 2)	0.20
KDQOL effect of disease	− 2 (− 5 to 1)	0.25
KDQOL SF-36 physical component summary	− 1 (− 4 to 1)	0.23
KDQOL SF-36 mental component summary	− 2 (− 4 to 0)	0.03
Renal biochemistry		
Serum bicarbonate (mmol/L)	1.1 (0.6 to 1.6)	< 0.001
Serum potassium (mmol/L)	0.0 (− 0.1 to 0.1)	0.80
eGFR (mL/min/1.73 m^2^)*	0.6 (− 0.8 to 2.0)	0.39
Serum creatinine (umol/L)*	− 8 (− 28 to 13)	0.46
Serum cystatin C (mg/L)*	− 0.01 (− 0.17 to 0.14)	0.89
Log [urinary albumin/creatinine ratio]	0.32 (− 0.05 to 0.70)	0.09
Cardiometabolic risk		
Log [NT-pro-BNP (pg/mL)]	0.13 (− 0.18 to 0.44)	0.42
Total cholesterol (mmol/L)	0.1 (− 0.2 to 0.3)	0.58
Systolic blood pressure (mmHg)	0 (− 4 to 3)	0.93
Diastolic blood pressure (mmHg)	1 (− 1 to 3)	0.16
HbA1c (mmol/mol)	1 (− 1 to 4)	0.38
Bone and mineral metabolism		
Log [TRACP-5b (IU/L)]	− 0.18 (− 0.43 to 0.08)	0.17
Log [Bs-ALP (μg/L)]	0.01 (− 0.11 to 0.13)	0.83
Log [PTH (pmol/L)]	0.03 (− 0.14 to 0.19)	0.75
Log [25OHD (nmol/L)]	− 0.08 (− 0.23 to 0.06)	0.24
1,25OHD (pmol/L) (SD)	3 (− 3 to 9)	0.30
Serum calcium (mmol/L)	0.02 (0.00 to 0.04)	0.11
Serum phosphate (mmol/L)	0.02 (− 0.03 to 0.06)	0.52
Other		
Haemoglobin (g/L)	− 0.1 (− 0.4 to 0.2)	0.48
Albumin (g/L)	0 (−1 to 1)	0.67
Log [TSH (mIU/L)]	0.07 (− 0.10 to 0.24)	0.39

*Repeated measures analyses using data from all available timepoints, adjusted for age and sex only

*1,25OHD* 1,25-dihydroxyvitamin D, *25OHD* 25-hydroxyvitamin D, *NT-pro-BNP* N-terminal pro B-type natriuretic peptide, *Bs-ALP* bone-specific alkaline phosphatase, *eGFR* estimated glomerular filtration rate, *HbA1c* glycosylated haemoglobin, *KDQOL* kidney disease quality of life, *PTH* parathyroid hormone, *SF-36* Short-form 36 questionnaire, *TRACP-5b* tartrate-resistant acid phosphatase 5b, *TSH* thyroid-stimulating hormone

Similar numbers of participants in each arm experienced at least one adverse event (131/152 [86.1%] in the bicarbonate arm vs 132/148 [89.1%] in the placebo arm; *p* = 0.38). More adverse events were recorded in the bicarbonate arm than in the placebo arm (457 vs 400) with an excess of events in the bicarbonate arm coded under gastrointestinal (45 vs 25), musculoskeletal (28 vs 17), cardiac (32 vs 19), nervous (24 vs 12) and respiratory (26 vs 14) systems. Full details are presented in Table 3. The difference in cardiac events was driven by an excess of myocardial infarction or acute coronary syndrome (10 vs 2), and not by a difference in decompensated heart failure (8 vs 10). Twenty-six deaths were recorded during the trial, with similar numbers in the bicarbonate and placebo arms (15 vs 11; *p* = 0.45). Time to event analysis showed no significant difference in the risk of death between groups (HR 1.30, 95% CI 0.60 to 2.83; *p* = 0.51).

**Table 3:** Adverse events by MedDRA System Order Class (SOC)

	Bicarbonate (*n* = 152)	Placebo (*n* = 148)
Number of adverse events per participant (%)		
0	21 (13.8)	16 (10.8)
1	23 (15.1)	41 (27.7)
2	27 (17.8)	35 (23.6)
3	35 (23.0)	15 (10.1)
4 or more	46 (30.3)	41 (27.7)
Total number of adverse events	457	400
SOC classification—number of events		
Blood and lymphatic system disorders	5	1
Cardiac disorders	32	19
Congenital, familial and genetic disorders	0	1
Ear and labyrinth disorders	1	1
Endocrine disorders	1	2
Eye disorders	6	6
Gastrointestinal disorders	45	25
General disorders and administration site conditions	14	20
Hepatobiliary disorders	0	0
Immune system disorders	0	0
Infections and infestations	113	118
Injury, poisoning and procedural complications	41	32
Investigations	5	7
Metabolism and nutrition disorders	19	27
Musculoskeletal and connective tissue disorders	28	17
Neoplasms benign, malignant and unspecified (including cysts and polyps)	9	16
Nervous system disorders	24	12
Psychiatric disorders	1	5
Renal and urinary disorders	23	23
Reproductive system and breast disorders	4	1
Respiratory, thoracic and mediastinal disorders	26	14
Skin and subcutaneous tissue disorders	16	11
Surgical and medical procedures	34	30
Vascular disorders	10	12

### Cost-effectiveness analyses

**Fig. 4:**
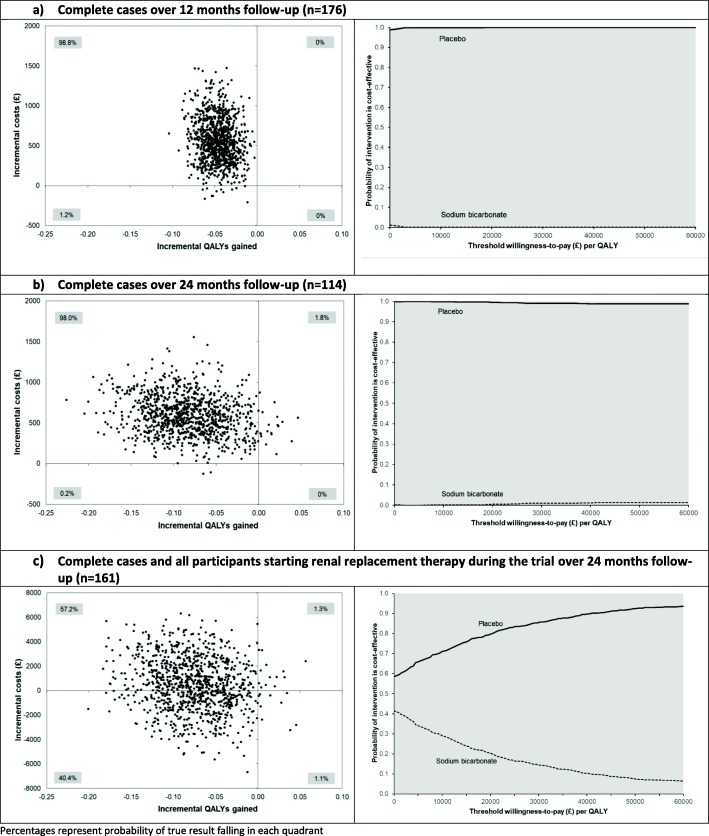
Scatterplot and cost-effectiveness acceptability curve of incremental cost difference and incremental QALY difference between randomised groups

In unadjusted analysis amongst complete cases, the mean costs per participant over the first 12 months of follow-up were £1234 amongst the bicarbonate group (*n* = 97) and £807 amongst the placebo group (*n* = 79). Table 4 shows the adjusted costs and QALYs for each group for complete cases at 12 month and 24 months, and imputed cases with renal replacement costs included. Costs were significantly higher amongst patients who received bicarbonate, and QALYs were significantly lower. The only exception to this was the imputed analysis, where cost differences were no longer significant. The robustness of these analyses was assessed using different acquisition cost estimates for bicarbonate therapy and inpatient stays, together with different estimates of benefit (ICECAP-O measure and global life satisfaction). Figure 4 shows the scatterplot and the associated cost-effectiveness acceptability curves for these analyses. Without including renal replacement costs, there is almost zero probability of the intervention being cost-effective at conventional thresholds of willingness to pay. With the inclusion of renal replacement costs, the probability that bicarbonate is cost-effective is no higher than 40%, and at the value of £30,000 per QALY conventionally used in the UK as a cost-effectiveness threshold, the probability is 14%. Across the 24-month follow-up, slightly more participants had at least one EQ-5D measure missing in the placebo group than in the bicarbonate group (45% vs 36%) and slightly more participants had at least one timepoint with missing costs in the placebo group than the bicarbonate group (36% vs 31%). However, the results were also robust to analyses that used multiple imputation for missing values.

**Table 4:** Adjusted (adjusted for baseline differences (age, gender, stage of CKD, baseline EQ-5D health utility score and baseline cost)) mean incremental costs, incremental QALYs/outcomes and incremental cost-effectiveness ratio between sodium bicarbonate vs placebo

Analysis	Incremental mean costs, £ (95% CI)^a, b, c^	Incremental mean QALYs/outcomes (95% CI)^a, b, c^	Mean incremental cost-effectiveness ratio (£/QALY/outcome)
Complete cases over 12-month follow-up (*n* = 176)	563.74 (88.18 to 1154.18)	−0.047 (− 0.078 to − 0.015)	Dominated
Sensitivity analysis: lower sodium bicarbonate cost^d^	352.76 (− 154.37 to 957.45)	− 0.047 (− 0.078 to − 0.015)	Dominated
Sensitivity analysis: lower inpatient stay cost^e^	539.03 (109.13 to 1050.45)	− 0.046 (− 0.078 to − 0.015)	Dominated
Sensitivity analysis: using ICECAP value^f^	636.20 (187.59 to 1189.24)	− 0.017 (− 0.032 to 0.0001)	Dominated
Sensitivity analysis: using life satisfaction value^g^	580.19 (143.38 to 1130.11)	− 0.396 (− 0.733 to − 0.059)	Dominated
Complete cases over 24-month follow-up (*n* = 114)^h^	591.00 (166.29 to 1078.36)	− 0.083 (− 0.166 to − 0.005)	Dominated
Sensitivity analysis: lower sodium bicarbonate cost^d^	242.59 (− 179.63 to 720.27)	− 0.083 (− 0.166 to − 0.005)	Dominated
Sensitivity analysis: lower inpatient stay cost^e^	593.74 (191.37 to 1072.07)	− 0.083 (− 0.166 to − 0.005)	Dominated
Sensitivity analysis: using ICECAP value^f^	598.87 (215.69 to 1052.43)	−0.051 (− 0.095 to − 0.010)	Dominated
Sensitivity analysis: using life satisfaction value^g^	682.44 (257.28 to 1142.63)	− 0.974 (− 1.762 to − 0.190)	Dominated
Complete cases over 24-month follow-up and all participants starting renal replacement therapy during the trial (*n* = 161)^h^	808.93 (− 4124.71 to 5411.89)	− 0.074 (− 0.151 to − 0.003)	Dominated
Sensitivity analysis: lower sodium bicarbonate cost^d^	534.61 (− 4385.90 to 5149.69)	− 0.074 (− 0.150 to − 0.003)	Dominated
Sensitivity analysis: lower inpatient stay cost^e^	817.21 (− 4097.90 to 5415.22)	− 0.073 (− 0.151 to − 0.001)	Dominated
Sensitivity analysis: using ICECAP value^f, i^	422.08 (− 4091.74 to 4629.60)	− 0.046 (− 0.090 to − 0.002)	Dominated
Sensitivity analysis: lower dialysis cost^j^	600.26 (− 3560.78 to 4379.06)	− 0.075 (− 0.154 to − 0.001)	Dominated
Sensitivity analysis: higher dialysis cost^k^	899.41 (− 4327.11 to 5714.04)	− 0.074 (− 0.156 to 0.002)	Dominated
Sensitivity analysis: using life satisfaction value^g, l^	928.18 (− 4373.23 to 5729.68)	− 0.072 (− 1.366 to 0.002)	Dominated

^a^Bootstrapped non-parametric 95% confidence interval (2.5th/97.5th percentile)

^b^Generalised linear model with γ distribution and power 0.65 link function to estimate incremental costs and ordinary least squares regression to estimate incremental QALYs (complete cases)

^c^Generalised linear model with Gaussian distribution and power 0.5 link function to estimate incremental costs and ordinary least squares regression to estimate incremental QALYs (114 complete cases + 47 participants starting renal replacement therapy during the trial with baseline EQ-5D). For incomplete cases, missing cost data were assumed to be zero and missing EQ-5D data were imputed by carrying forward the last observation

^d^Applied average cost of three generic sodium bicarbonate 500 mg with the lowest price, £0.14/day

^e^Applied average of lower quartile unit cost for non-elective inpatient and elective inpatient bed days, £287/day

^f^Adjusted for baseline differences (age, gender, stage of CKD, baseline ICECAP value and baseline cost)

^g^Adjusted for baseline differences (age, gender, stage of CKD, baseline life satisfaction value and baseline cost)

^h^Discounted at 3.5% per year

^i^Two participants from the bicarbonate group without any ICECAP data were excluded from the analysis, *n* = 159

^j^Applied average of lower quartile unit cost for haemodialysis (£134/visit) and peritoneal dialysis (£66/visit)

^k^Applied average of upper quartile unit cost for haemodialysis (£180/visit) and peritoneal dialysis (£77/visit)

^l^Two participants (1 from each group) without any life satisfaction data were excluded from the analysis, *n* = 159

*Abbreviations*: *QALYs* quality-adjusted life years, *ICER* incremental cost-effectiveness ratio, *SA* sensitivity analysis, *ICECAP* Investigating Choice Experiments for the preferences of older people CAPability tool, *CKD* chronic kidney disease

## Discussion

### Key findings

Our study is the first to examine the effect of oral bicarbonate therapy on physical function, health-related quality of life and healthcare costs in older people with advanced CKD. In this trial, administration of oral sodium bicarbonate using a dose regime similar to that currently used in UK practice did not improve physical function or quality of life, increased adverse events and had no impact on the rate of progression of CKD compared to placebo in older people with CKD category 4 or 5 and serum bicarbonate concentrations of < 22 mmol/L. Consistent with these findings, health economic analysis showed that bicarbonate was less cost-effective than placebo.

### Strengths and weaknesses

Important strengths of this trial were its large size in comparison to previous trials in this area, the fact that it enrolled the age group most likely to suffer the consequences of CKD; clinically relevant follow-up time; participant, clinician and researcher masking; and broad inclusion criteria. In contrast to almost all previous trials, our use of a placebo control reduced the opportunities for bias. An additional strength was the broad range of outcome measures examined, with a particular focus on physical function and quality of life. These are the outcomes that older people report being most important to them, and this focus is of particular importance in this group of patients with extensive multimorbidity. A narrow focus on a single disease—even in patients with advanced CKD—may not be helpful for holistic decision-making; considering physical function and quality of life enables an assessment of the overall benefit of treatment to patients in a way that organ-specific measures do not.

A number of limitations require further comment. Given the modest increase in serum bicarbonate seen with the dosing schedule used in this trial, it is possible that larger doses of oral bicarbonate are required to increase serum bicarbonate in older people with CKD, although the mechanisms that might underpin such a hypothesis are unclear. Whilst such an approach would be of mechanistic interest, we do not believe that higher doses (more than the six tablets per day) would be well tolerated by older people; the adherence rate would likely be even lower than the moderate adherence rate observed in the current trial. The higher rate of adverse events in the bicarbonate arm, particularly gastrointestinal adverse events, slightly lower physical function measures and lack of a relationship between adherence and treatment effect size, all argue that a higher dose of bicarbonate is unlikely to produce health benefits in this patient group. We cannot exclude a potentially beneficial effect of bicarbonate in different groups of patients with CKD, however. In particular, the current trial enrolled an overwhelmingly white European population. CKD of different aetiologies may respond differently and may explain some of the heterogeneity seen in one recent systematic review [9, 27].

The original target for recruitment for this trial (380 participants) was not reached despite recruiting from 27 UK sites. This was in part due to a lack of clinical equipoise; surveys performed by the trial team during the trial suggested that most UK nephrologists were treating patients with serum bicarbonate concentrations < 22 mmol/L with bicarbonate already, thus reducing the pool of eligible participants. It is possible that those most likely to respond to bicarbonate supplementation were already taking bicarbonate as part of routine clinical practice, thus further diluting the effect of the intervention. The dropout rate in the trial was considerable and slightly higher than the 10% per 6-month follow-up that we anticipated. Such a dropout rate is not unexpected given the high levels of multimorbidity and frailty in this patient population. Although dropout reduces the power of the trial to detect significant treatment effects, this is mitigated to some extent by our use of mixed models with repeated measures for the secondary analyses, which use all available data and effectively impute missing values. Thus, despite the lower than planned sample size, and the lower than anticipated availability of primary outcome data at 12 months, our results exclude the minimum clinically important improvement in the primary outcome with a high degree of confidence. Some investigators have proposed a smaller minimum clinically important difference of 0.5 points for the SPPB [28]; our results also exclude this improvement with a high degree of confidence. Although the trial was designed to have adequate power for the primary outcome and for the EQ-5D outcome, it was not powered to detect clinically important differences in the rate of deterioration of renal function, or for rates of commencing dialysis. We were unable to include the costs of every other medication used in the cost-effectiveness analysis, which could potentially lead to over- or underestimation of cost-effectiveness. Given the higher number of adverse events in the bicarbonate group (which would be expected to require additional drugs to treat), it is more likely that additional medication costs would be higher in the bicarbonate arm, further reducing the likelihood that bicarbonate is cost-effective.

### Strengths and weaknesses in relation to other studies

Given the association between metabolic acidosis and a range of adverse outcomes seen in CKD, it would be expected that amelioration of acidosis (i.e. increasing serum bicarbonate) could improve physical function, lessen deterioration in renal function and improve measures of cardiovascular health and bone and mineral metabolism in patients with CKD. A recent systematic review [9] found no randomised controlled trial evidence around the effect of bicarbonate on physical function or quality of life; the current trial therefore provides an important test of the effect of bicarbonate on these important patient-centred outcomes. A previous single-centre trial of bicarbonate versus usual care found progressive increases in serum bicarbonate over a 2-year period in the bicarbonate group; measures of anthropometry were better in the bicarbonate group, and fewer participants progressed to end-stage renal disease in the bicarbonate group (4/67 vs 22/67) [27]. However, this trial did not use placebo, and thus, healthcare providers and participants were aware of group allocation. It is possible that the lack of masking could inflate the effect size seen in this trial, perhaps by an impact on decision-making around renal replacement and on other treatment decisions driven by knowledge of whether a participant was receiving bicarbonate. Participants in this previous trial were recruited from a single centre and were much younger (mean age 55 years), and a high percentage were of south Asian or black origin.

In our trial, oral bicarbonate produced only a modest increase in serum bicarbonate concentration relative to placebo; this difference was maximal at 3 months and converged with the placebo group by 24 months. Bicarbonate concentrations in the placebo group rose gradually over time, which again limited the contrast between the two groups; this may be due in part to the pragmatic nature of the trial design, where physicians were free to switch participants to bicarbonate therapy if this was felt to be clinically indicated. The effect seen in both recent systematic reviews [9, 10] was greater than that observed in the current trial, with a mean 3 mmol/L higher serum bicarbonate in the treatment arm compared to control by the end of follow-up; restricting analyses to 1-year follow-up gave similar results. It is likely that the combination of a modest dose of bicarbonate (in comparison to some other trials) and suboptimal adherence contributed to this finding, but as this dosing regimen reflects current UK practice, patients in the real world are likely to sustain similarly modest increases in serum bicarbonate concentrations. A recent dose-ranging pilot trial (the BASE trial) suggested that higher dose (approximately 5 g/day) of bicarbonate was more effective than lower dose (approximately 3 g/day) of bicarbonate in increasing serum bicarbonate concentrations in patients with CKD 3 or 4; the higher dose provided an additional 1.3-mmol/L increase in mean serum bicarbonate concentration compared to the lower dose [29]. Most trials included in the systematic review used a ‘treat-to-target’ approach. This would allow participants with lower bicarbonate concentrations to receive higher doses but could also potentially increase adverse events. Although previous small trials have used such an approach successfully without evidence of significant harm [30], harms have not been well reported in bicarbonate trials to date, and our more comprehensive approach to adverse event reporting revealed an excess of events in the bicarbonate arm. Our ability to titrate doses to the levels used in some previous trials was limited; we made the decision at the design stage to limit titration to doubling of the dose at 3 months, which was performed only if serum bicarbonate concentrations were still < 22 mmol/L. Although this decision was taken to reflect the state of usual UK bicarbonate prescribing practice, practice is heterogeneous and higher doses of bicarbonate may have greater effects for some patients. In addition, for participants in the treatment group with only modest degrees of acidosis, only a small improvement in serum bicarbonate was required to cross this threshold, which limited the number of participants for whom a higher dose of bicarbonate was prescribed.

Although no studies have studied the association between serum bicarbonate levels and our primary outcome of the Short Physical Performance Battery, observational data suggest a 7–8% increase in incident functional limitation for each 1 mmol/L lower bicarbonate level in a cohort of older people, although few of this cohort had CKD [7]. Similarly, participants in NHANES with a serum bicarbonate concentration < 23 mmol/L had a 14% higher risk of low gait speed for every 1 mmol/L lower bicarbonate concentration [6]. These results are not incompatible with our trial results; larger increases in serum bicarbonate concentration may be required to produce clinically meaningful changes in physical performance measures or biochemical parameters such as bone metabolism markers.

Most trials in the systematic review did not use placebo. The unmasked nature of these trials is likely to have contributed to the larger treatment effects observed and may also have influenced decision-making about the commencement of renal replacement therapy in some trials. Differential dropout is unlikely to explain the small difference we observed in the current trial as dropout numbers at each timepoint were similar in each arm. Although adherence was not optimal in the current trial, the dose of bicarbonate prescribed was similar to that used in a previous trial [25] that observed greater increases in serum bicarbonate, and both the dose used and the adherence levels observed in our trial are likely to reflect those seen in clinical practice, especially amongst older people taking a large number of medications. A modest difference in eGFR at both 1 year and at the end of follow-up was seen in the recent systematic review, although too few data were available to calculate the mean rate of decline in eGFR. No significant treatment effect on blood pressure or body weight was seen in the systematic review, and our findings are consistent with these observations.

A number of other trials of bicarbonate therapy are currently in progress or have just been published [31–33]. These trials target a range of severities of CKD (categories 3b to 5), a range of entry serum bicarbonate concentrations and for two of the trials, a strategy of dose adjustment to keep serum bicarbonate > 24 mmol/L is employed. None of these trials targets older people as a specific group however. The large UBI trial, based in Italy, randomised 795 individuals with CKD stage 3b or 4 to usual care or a strategy of bicarbonate treatment to a target of 24–28 mmol/L. The mean dose required to reach this serum bicarbonate concentration (some 3–4 mmol/L higher than in the usual care arm) was 6 g/day of sodium bicarbonate—much higher than used in the BiCARB trial. The UBI trial showed a significant reduction in the rate of progression of CKD, death and dialysis commencement in the bicarbonate arm compared to usual care [34]. Patients in this trial were slightly younger (mean age 67 years) and had considerably better renal function (mean eGFR of 36 mL/min/1.73 m^2^), but the trial was not placebo-controlled or blinded. In contrast, a smaller (*n* = 149) placebo-controlled randomised trial in patients with CKD stages 3 and 4 with similar baseline eGFR found a 1–2-mmol/L increase in serum bicarbonate concentration in the treatment arm compared to placebo, using a dose equivalent to between 2 and 2.5 g per day of sodium bicarbonate [32]. Similar to the findings of the BiCARB trial, there was no effect of bicarbonate supplementation on eGFR, muscle function, blood pressure or quality of life.

Recent trial data suggests that the new hydrochloric acid binder veverimer can successfully increase serum bicarbonate concentrations; a mean 3-mmol/L increase compared to placebo was observed over 12 weeks [35]. In addition, veverimer improved disease-specific quality of life measured using the KDQoL tool, and improved chair stand time relative to placebo over 1 year of treatment [36], albeit in a younger population (mean age 61 years) than studied in the current trial.

## Conclusions

### Implications and future research

Our results suggest that at least for patients aged 60 and over with CKD GFR categories 4 and 5, 1.5 to 3 g per day of oral bicarbonate does not produce any health benefits and may be associated with net harms. Whilst other indications for control of acidosis exist (for example high potassium concentrations), evidence from the current trial suggests that the additional cost, treatment burden and side effects of oral bicarbonate may not justify its use in older people with advanced CKD and mild degrees of metabolic acidosis (i.e. serum bicarbonate concentration < 22 mmol/L). Raising bicarbonate levels by an amount sufficient to produce useful clinical effects will require larger doses than we used in this trial and is likely to require a ‘treat-to-target’ strategy. However, such doses may not be tolerated by many older people. Alternative strategies, such as the use of hydrochloric acid binders, may provide a way round this issue, but such agents need to be tested against current practice in representative groups of patients, using a range of outcomes relevant to older people including physical function, quality of life and deterioration of renal function.

## Supplementary information

**Additional file 1.** Comparison of baseline characteristics of participants completing and not completing SPPB (primary outcome) at 12 months.

## Data Availability

Deidentified, individual participant-level data are available to bona-fide academic research teams, subject to submission of an outline of the purpose for which it will be used, and subject to approval by an independent Data Access Committee process hosted by the trial Sponsor (University of Dundee). Please contact the corresponding author or the Sponsor (TASCgovernance@dundee.ac.uk).

## Abbreviations

CI: Confidence interval
CKD: Chronic kidney disease
DMC: Data Monitoring Committee
eGFR: Estimated glomerular filtration rate
EQ-5D: EuroQoL 5 dimension tool
ICECAP-O: ICEpop CAPability measure for Older people
KDQoL: Kidney Disease Quality of Life
MDRD4: Modified Diet in Renal Disease 4 variable equation
NHANES: National Health and Nutrition Examination Survey
NHS: National Health Service
NIHR: National Institute for Health Research
QALY: Quality-adjusted life years
SPPB: Short Physical Performance Battery
TSC: Trial Steering Committee
