# Supplementary material for: Clinical and cost-effectiveness of oral sodium bicarbonate therapy for older patients with chronic kidney disease and low-grade acidosis (BiCARB): a pragmatic randomised, double-blind, placebo-controlled trial
Source: BMC Med. 2020 Apr 9;18:91. doi: 10.1186/s12916-020-01542-9 (PMC7144058; doi:10.1186/s12916-020-01542-9)
Supplement: Supplementary file 1 — Additional file 1. Comparison of baseline characteristics of participants completing and not completing SPPB (primary outcome) at 12 months. [file 12916_2020_1542_MOESM1_ESM.docx]

**Additional File 1**

**Comparison of baseline characteristics of participants completing and not completing SPPB (primary outcome) at 12 months**

|  | Bicarbonate | | | Placebo | | |
| --- | --- | --- | --- | --- | --- | --- |
|  | Completed SPPB at 12 months | Did not complete SPPB at 12 months | p | Completed SPPB at 12 months | Did not complete SPPB at 12 months | p |
| SPPB (SD) | 8.3 (2.4) | 7.4 (2.2) | 0.04 | 8.5 (2.0) | 7.6 (2.3) | 0.01 |
| Handgrip strength (kg) (SD) | 25.3 (9.8) | 20.4 (7.5) | 0.002 | 25.5 (8.6) | 22.6 (8.8) | 0.05 |
| Six minute walk distance (m) (SD) | 344 (119) | 235 (132) | <0.001 | 343 (113) | 277 (152) | 0.006 |
| eGFR (ml/min/1.73m^2^) (SD) | 21.5 (6.4) | 19.1 (7.3) | 0.04 | 19.6 (6.4) | 18.4 (7.3) | 0.30 |
| Serum bicarbonate (mmol/L) (SD) | 20.1 (2.2) | 20.2 (2.6) | 0.94 | 20.1 (2.4) | 19.6 (2.6) | 0.21 |

SPPB: Short Physical Performance Battery. eGFR: estimated Glomerular Filtration Rate
